# A Label-Free Electrochemical Immunosensor for CEA Detection on a Novel Signal Amplification Platform of Cu_2_S/Pd/CuO Nanocomposites

**DOI:** 10.3389/fbioe.2021.767717

**Published:** 2021-12-10

**Authors:** Linlin Cao, Wen Zhang, Sumei Lu, Chengjie Guo, Peijun Wang, Dantong Zhang, Wanshan Ma

**Affiliations:** ^1^ Department of Laboratory Medicine, Shandong Provincial Qianfoshan Hospital, Shandong University, Jinan, China; ^2^ Department of Clinical Laboratory, Zibo Central Hospital, Shandong University, Zibo, China; ^3^ Department of Laboratory Medicine, The First Affiliated Hospital of Shandong First Medical University, Jinan, China

**Keywords:** carcinoembryonic antigen, Cu_2_S/Pd/CuO, immunosensor, colorectal cancer, label-free

## Abstract

Carcinoembryonic antigen (CEA) is regarded as one of the crucial tumor markers for colorectal cancer. In this study, we developed the snowflake Cu_2_S/Pd/CuO nanocomposite to construct an original label-free electrochemical immunosensor for the ultrasensitive detection of CEA levels. The nanocomposite of cuprous sulfide (Cu_2_S) with Pd nanoparticles (Pd NPs) was synthesized through an *in situ* formation of Pd NPs on the Cu_2_S. Cuprous sulfide (Cu_2_S) and CuO can not only be used as a carrier to increase the reaction area but also catalyze the substrate to generate current signal. Palladium nanoparticles (Pd NPs) have excellent catalytic properties and good biocompatibility, as well as the ability of excellent electron transfer. The immunosensor was designed using 5 mmol/L H_2_O_2_ as the active substrate by optimizing the conditions with a detection range from 100 fg/ml to 100 ng/ml and a minimum detection limit of 33.11 fg/ml. The human serum was detected by electrochemical immunoassay, and the results were consistent with those of the commercial electrochemical immunosensor. Therefore, the electrochemical immunosensor can be used for the detection of human serum samples and have potential value for clinical application.

## Introduction

Colorectal cancer (CRC) is one of the most frequent malignancies worldwide and is correlated with high mortality (Dekker et al., 2019). According to the latest statistics of the 2020 Global Cancer Statistics Report, there were 1,880,725 new cases of CRC. Colorectal cancer morbidity ranks third among malignancies, but second in terms of mortality (Sung et al., 2021). Carcinoembryonic antigen (CEA) is used as an important indicator for the diagnosis, treatment, recurrence, and metastasis of CRC (Konishi et al., 2018; Odeny et al., 2020). Additionally, CEA was also associated with other tumors, such as lung cancer (Grunnet and Sorensen, 2012), breast cancer (Tang et al., 2016), and pancreatic cancer (Xing et al., 2018). Therefore, it is essential to establish a rapid, sensitive, and reliable method for detecting CEA.

Currently, several assays have been applied to detect tumor markers in clinical practice, including enzyme-linked immunosorbent assay (ELISA) (Overholt et al., 2016; Zhang et al., 2019), electrochemiluminescence immunoassay (ECLI) (Wei et al., 2017; Nie et al., 2018), electrochemical immunosensor (Yan et al., 2019; Xiang et al., 2020; Biswas et al., 2021; Jian et al., 2021), and radioimmunoassay (Poncelet et al., 2010; Kawamoto et al., 2019). Electrochemical immunosensors are biosensing devices that convert biochemical reactions into electrical signals based on the combination of highly sensitive sensing technology and specific immune reactions to study the reaction kinetics of antigens and corresponding antibodies. They have the advantages of high specificity and sensitivity, rapidity, low cost, and simple operation (Cho et al., 2018; Felix and Angnes, 2018). Particularly, label-free immunosensors directly detect the signal changes of the antigen–antibody complex, which greatly simplifies the sensor preparation and operation and does not require secondary antibody markers (Filik and Avan, 2019; Tan et al., 2020; Rahmati et al., 2021).

Nanomaterials, such as graphene oxide, metal nanoparticles, and metal–organic frameworks (MOFs), are often used as a means of signal amplification to heighten the sensitivity of sensors because of their high specific surface area, prominent electron transfer ability, and excellent biocompatibility (Song et al., 2016; Farka et al., 2017). Among them, metal nanomaterials (Chen et al., 2021; Simsek and Wongkaew, 2021) have attracted strong attention because of their stronger electrical conductivity, excellent catalysis, larger specific surface area, and convenient control. Palladium nanoparticles (Pd NPs) have efficient catalytic activity toward hydrogen peroxide substrates (Edwards et al., 2014; Trujillo et al., 2021) and are excellent materials for the construction of immunosensors (Han et al., 2020). However, when Pd NPs are exposed to a relatively harsh electrochemical environment, their stability worsens, resulting in the dissolution and migration of surface Pd atoms, which leads to the agglomeration of nanoparticles and the reduction of the surface area (Gao et al., 2019; Ma et al., 2020). Therefore, the noble metal materials of Pd are usually dispersed on the carrier to obtain nanocomposites with better biocompatibility, higher electrical conductivity, and more excellent catalytic performance.

In recent years, many researchers have focused on using semiconductor materials as carriers of noble metals to improve their catalytic performance, including metal oxides, MoS_2_, and MOFs (Yang et al., 2017; Feng et al., 2018; Yusuf et al., 2021). The snowflake cuprous sulfide (Cu_2_S), which has an exceptionally high surface area, is considered to be a potential support material to load Pd NPs. Herein, Cu_2_S/Pd synthesized by *in situ* growth exhibiting satisfactory stability showed good catalytic performance and catalysis for hydrogen peroxide (H_2_O_2_) reduction (Zhang et al., 2018; Gao et al., 2020). Interestingly, we found that when Cu_2_S was partly oxidized to CuO, the resulted Cu_2_S/Pd/CuO nanocomposite possessed a more excellent catalytic performance, which could be a preferred signal amplification platform for the fabrication of immunosensors.

In this study, a novel composite material of Cu_2_S/Pd/CuO was synthesized and used to construct a label-free electrochemical immunosensor for CEA sensing, achieving high sensitivity, a wide detection range, and a low detection limit, and was validated in the analysis of human serum samples. Therefore, the proposed immunosensor has great potential for clinical application.

## Materials and Methods

### Reagents and Equipments

Ethylenediamine (EDA, CAS no. 107-15-3), CuCl_2_ 2H_2_O (CAS no. 10125-13-0), (NH_2_)_2_CS (CAS no. 62-56-6), and Na_2_PdCl_4_ (CAS no. 13820-53-6) were purchased from Macklin Biochemical Co., Ltd. (Shanghai, China). Bovine serum albumin (BSA) (CAS no. 9048-46-8; storage, 2–8C), CEA (L2C01001, 2023-12; 1 mg/ml, −20C), and CEA antibody (L1C00202, 2023-12; 1.9 mg/ml, −20C) were from Shanghai Lingchao Biological New Material Technology Co., Ltd. (Shanghai, China). Phosphate buffer solutions (PBS) were prepared with Na_2_HPO_4_ (CAS no. 7558-79-4) and KH_2_PO_4_ (CAS no. 7778-77-0). Human serum was obtained from Zibo Central Hospital. Ultrapure water (18.25 Ω) was made in the laboratory throughout the experiments. Hydrogen peroxide (H_2_O_2_, 30 wt%) was purchased from Shuangshuang Chemical Co., Ltd. (Yantai, China).

The electrochemical measurements were performed on the electrochemical workstation (China). Conventional three-electrode systems used in electrochemical measurement include glass carbon electrode (GCE), saturated calomel electrode, and platinum wire electrode. The Tecnai G2 F20 transmission electron microscope (Hillsboro, OR, USA) was used for transmission electron microscopy (TEM) image acquisition. The JEOL JSM-6700F microscope (Tokyo, Japan) was used to record the X-ray energy (EDX) spectrum. SEM images were taken using the FEI Quanta FEG250 Field Emission Environmental Scanning Electron Microscope (Hillsboro, OR, USA).

### Preparation of Cu_2_S

The material preparation of Cu_2_S was consistent with that reported in the literature (Zhang et al., 2018), and the method of hydrothermal synthesis was adopted. Firstly, 1 mmol CuCl_2_ 2H_2_O was dissolved in 30 ml EDA and 3 mmol thiourea was added. Then, the mixture was stirred with a magnetic mixer for 2 h at room temperature. After stirring, the mixed solution was transferred into a 50-ml polytetrafluoroethylene (PTFE) lined autoclave and reacted at 80°C for 8 h. Finally, the centrifugal Cu_2_S was washed with anhydrous ethanol and secondary deionized water and then dried in a freeze dryer for the next step.

### Preparation of Cu_2_S/Pd

Polyvinylpyrrolidone (PVP, 50 mg) and synthesized Cu_2_S (7.2 mg) were added into 8 ml secondary deionized water. Then, 5 ml of 10 mmol/L Na_2_PdCl_4_ solution was added to the above solution. Subsequently, the mixture was stirred with a magnetic mixer for 20 min and washed with ethanol and secondary deionized water three times. Finally, the obtained black powdery Cu_2_S/Pd was dried in vacuum.

### Preparation of Cu_2_S/Pd/CuO

The GCE (0.4 µm in diameter) was first polished with 0.05 µm aluminum oxide powder and then thoroughly rinsed with ultrapure deionized water to acquire a fresh and transparent surface. The oxidation peak of bare GCE is less than 100 mV with the reduction peak. The polished GCE was covered with deionized water to prevent oxidation, and then the electrode was blown dry with ear washers. Afterwards, the GCE electrode was modified with Cu_2_S/Pd (6 μl, 2 mg/ml) and the three-electrode system was assembled. The electrode was placed into 10 ml PBS, and 40 μl H_2_O_2_ (5 M) was injected into PBS under the operation of chronoamperometry. Finally, the residual H_2_O_2_ on the surface of the working electrode was washed off with PBS and the modified electrode dried.

### Preparation of Electrochemical Immunosensor

**FIGURE 1:**
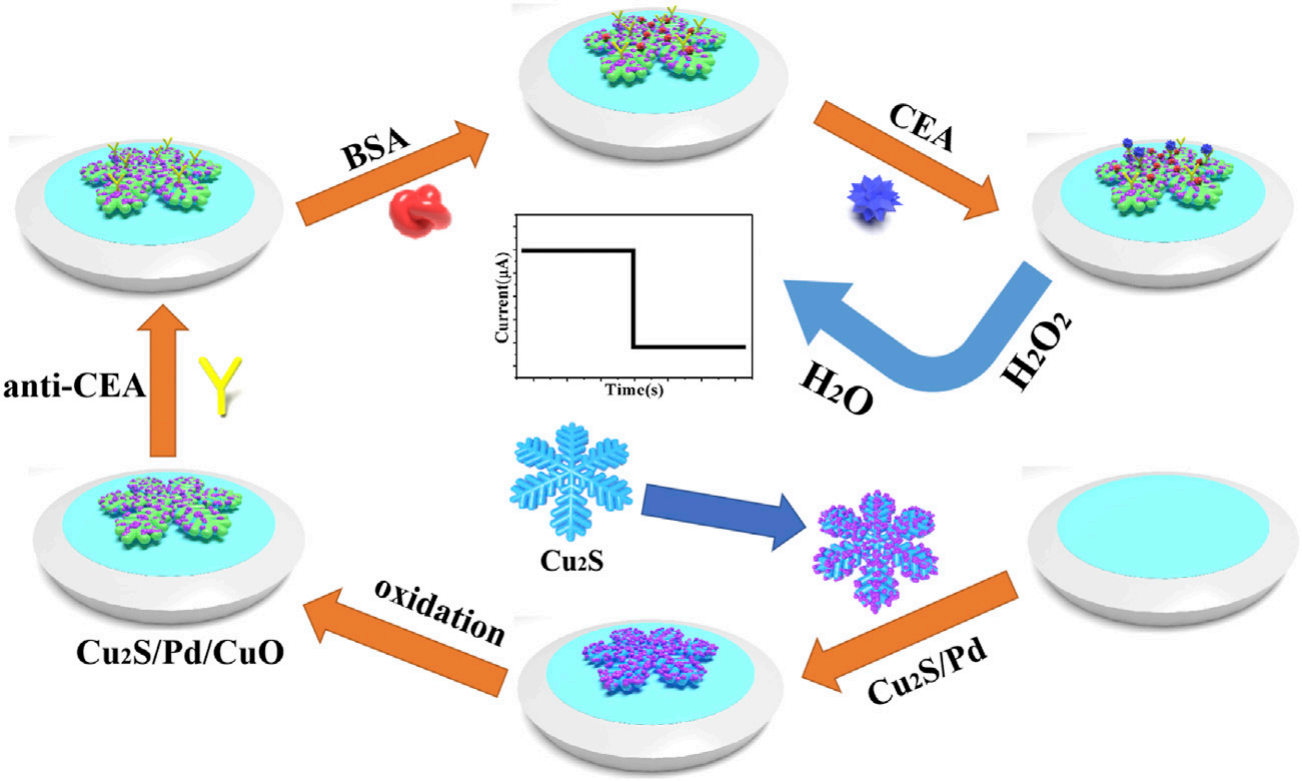
Construction process of the electrochemical immunosensor for carcinoembryonic antigen (CEA) detection.

To illustrate the whole process of the experiment more clearly, Figure 1 shows the layered self-assembly process of the immune sensor. The CEA antibody (anti-CEA, 6 μl, 2 mg/ml) was added to the surface of Cu_2_S/Pd/CuO/GCE and incubated at 4C. After washing with PBS (pH 6.81), bovine serum albumin (BSA) solution (1 wt%, 3 μl) covered the anti-CEA/Cu_2_S/Pd/CuO/GCE to block nonspecific active sites between the substrate nanocomposites and CEA. After 60 min of incubation, the BSA/anti-CEA/Cu_2_S/Pd/CuO/GCE was washed with PBS (pH 6.81) and was added different concentration gradients of CEA (from 6 μl, 100 fg/ml to 100 ng/ml) for 60 min to optimize the reaction conditions between CEA and anti-CEA. Finally, the prepared working electrodes were washed with PBS (pH 6.81) and stored at 4°C.

### Electrochemical Measurements

The electrochemical measurement was carried out using electrochemical workstation CHI760E. The immunosensor uses −0.4 V as the scanning potential to measure the current curve of the ampere. After the background current remained stable, H_2_O_2_ (5 M, 10 μl) was emptied into PBS (10 ml, pH 6.81), stirred with the magnetic stirrer, and the changes of the current response were recorded. The cyclic voltammetry (CV) test was carried out in K_3_ [Fe(CN)_6_^3−^] solution (5 mM). Under the open circuit voltage of 0.196 V, electrochemical impedance spectroscopy (EIS) analysis was performed for the potassium ferricyanide solution, and a Nernst plot was drawn to record each fixed step. All the electrochemical measurement processes were conducted at room temperature.

## Results and Discussion

### Characterization of Cu_2_S, Cu_2_S/Pd, and Cu_2_S/Pd/CuO

**FIGURE 2:**
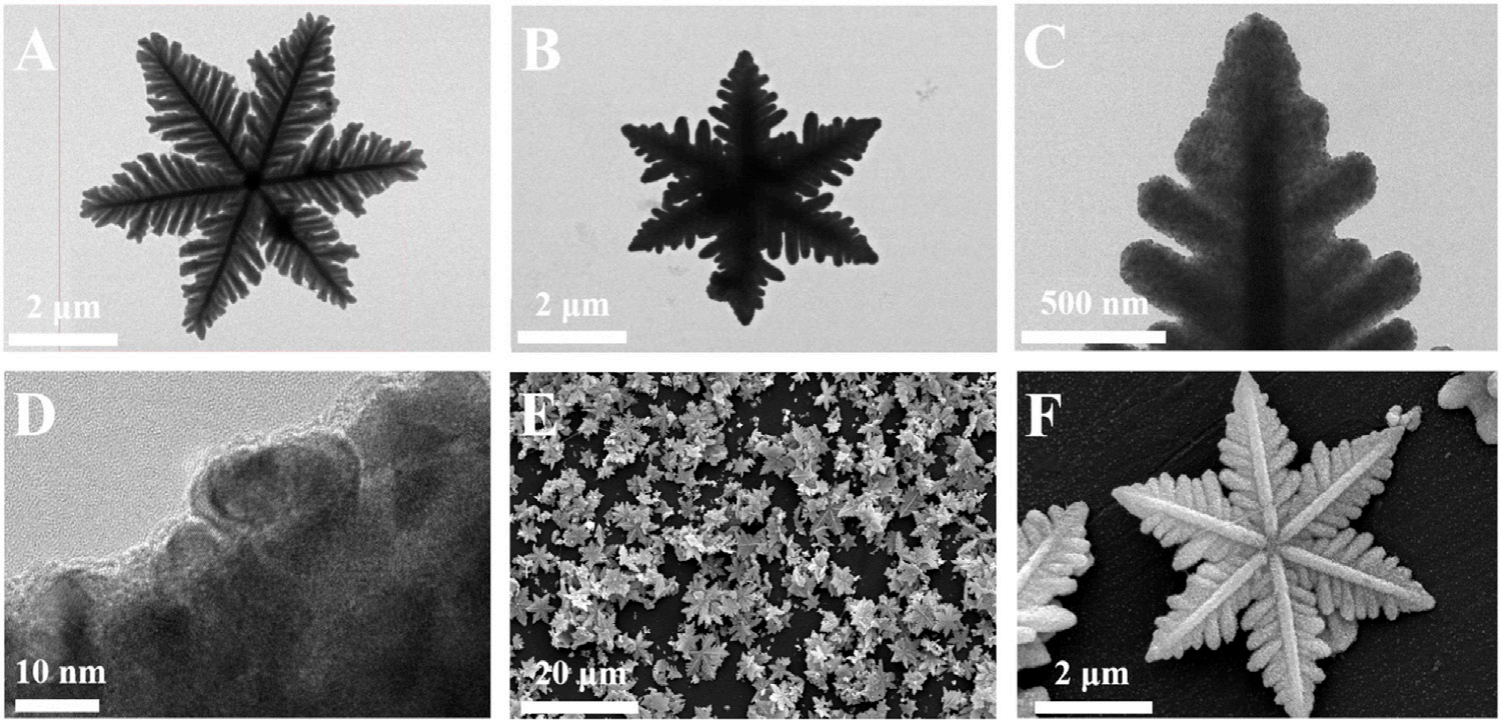
**(A–D)** TEM images of Cu_2_S **(A)** and Cu_2_S/Pd **(B–D)**. **(E–F)** SEM images of Cu_2_S/Pd/CuO.

**FIGURE 3:**
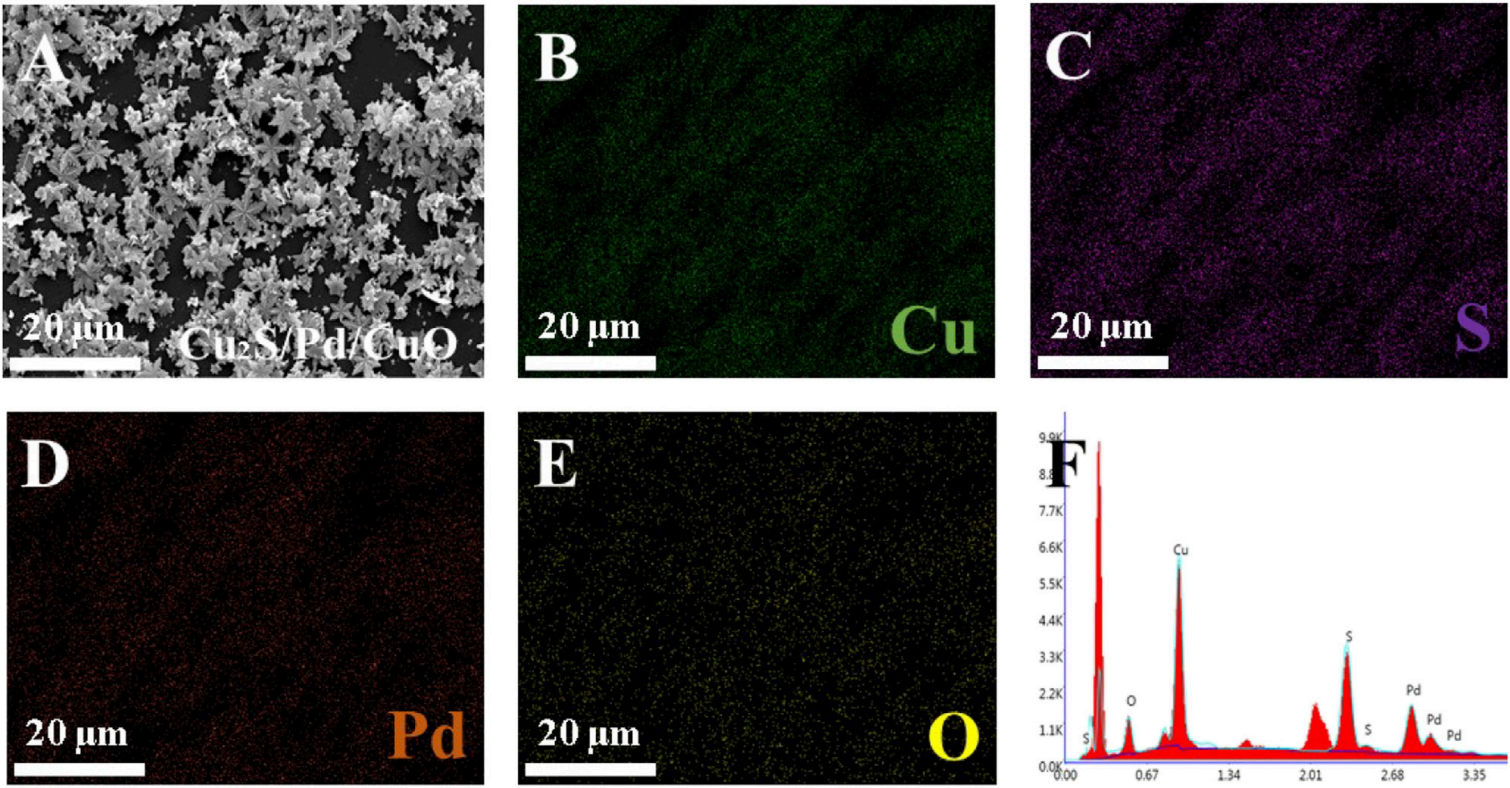
Characterization of Cu_2_S/Pd/CuO nanocomposites. **(A)** SEM image of Cu_2_S/Pd/CuO. **(B–F)** Elemental mappings of Cu **(B)**, S **(C)**, Pd **(D)**, and O **(E)**. **(F)** X-ray energy (EDX) spectrum of Cu_2_S/Pd/CuO.

Figure 2 shows the morphology of the prepared material. It can be found that the obtained Cu_2_S is snowflake-shaped and has a flat symmetrical structure with six orientated petals radially extending from the central button; it has a diameter of 4–6 um based on the TEM image (Figure 2A). When Pd NPs wrapped around Cu_2_S, they caused the Cu_2_S/Pd to have an unclear boundary line and increased the specific surface area, as shown in Figures 2B–D. In Figures 2E, F, the successful preparation of Cu_2_S/Pd/CuO nanocomposites was confirmed by TEM. The mapping spectra of Cu, S, Pd, and O (Figures 3B–E, respectively) clearly indicated that the distribution of the four elements was relatively uniform, specifying that the material is well constructed. At the same time, the EDX spectra (Figure 3F) confirmed that the composite material contained the Cu, S, Pd, and O elements.

### Electrochemical Characterization

**FIGURE 4:**
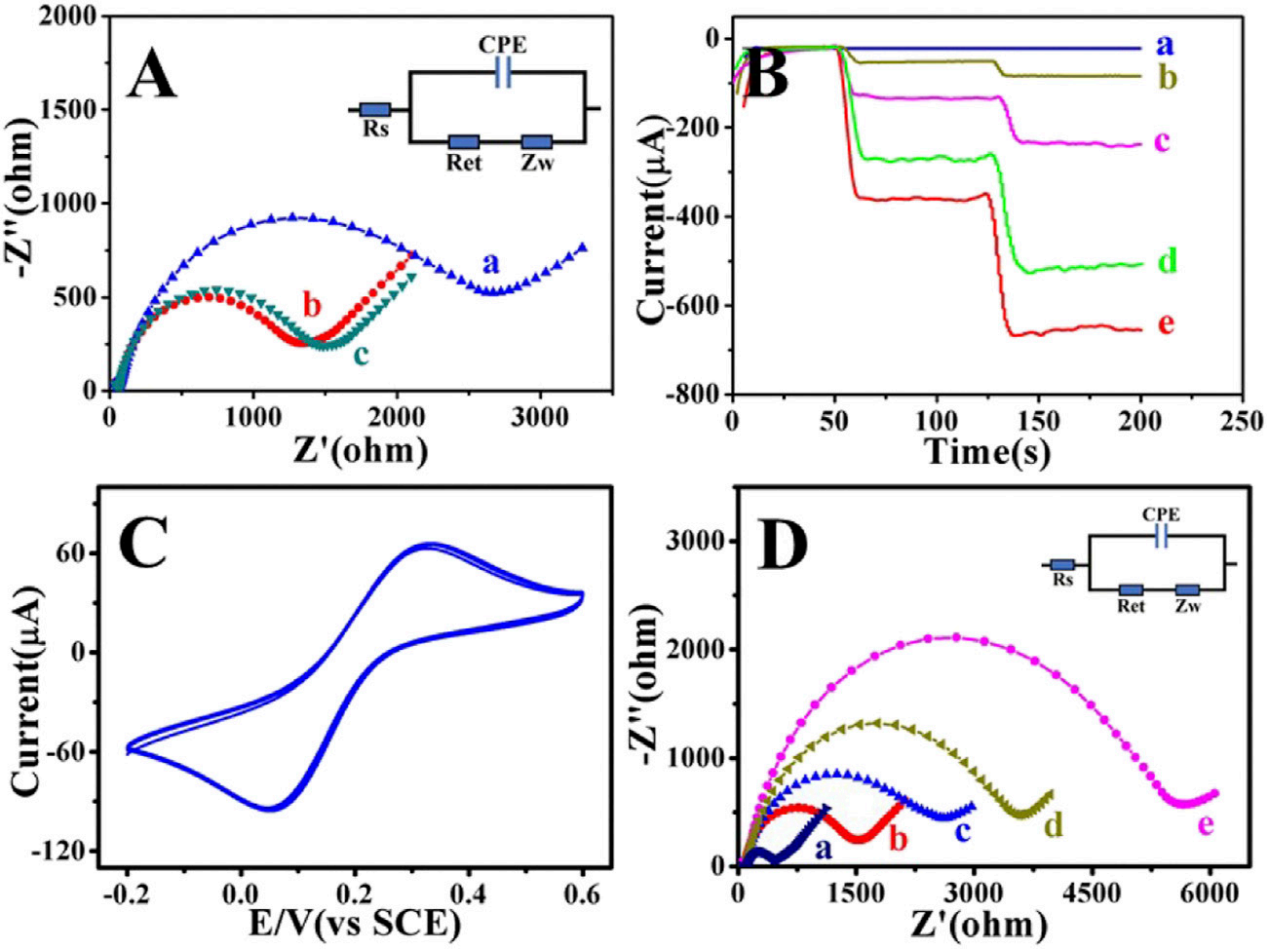
Electrochemical characterization of Cu_2_S/Pd/CuO nanocomposites. **(A)** Electrochemical impedance spectroscopy (EIS) of Cu_2_S (*a*), Cu_2_S/Pd (*b*), and Cu_2_S/Pd/CuO (*c*). **(B)** Analysis of the *i*–*t* (current–time): glass carbon electrode (GCE) (*a*), Cu_2_S (*b*), Cu_2_S/CuO (*c*), Cu_2_S/Pd (*d*), and Cu_2_S/Pd/CuO (*e*). **(C)** Cyclic voltammetry (CV) diagram of Cu_2_S/Pd/CuO-modified GCE. **(D)** EIS of GCE (*a*), Cu_2_S/Pd/CuO/GCE (*b*), anti-CEA/Cu_2_S/Pd/CuO/GCE (*c*), BSA/anti-CEA/Cu_2_S/Pd/CuO/GCE (*d*), and CEA/BSA/anti-CEA/Cu_2_S/Pd/CuO/GCE (*e*). *BSA*, bovine serum albumin; *CEA*, carcinoembryonic antigen.

EIS can be used to compare the electrical conductivity of different materials (Strong et al., 2021). In this work, EIS was used to monitor the change of electron transfer resistance (*R*_et_). The semicircle part represents the electron transfer limitation. The larger the semicircle diameter, the greater the *R*_et_. Cu_2_S (curve a) has poor electrical conductivity, which was obviously enhanced after palladium atoms were loaded and can be used as a substrate material. The electrical conductivity of Cu_2_S/Pd/CuO (curve c) was basically the same as that of Cu_2_S/Pd (curve b), as shown in Figure 4A and Supplementary Table S1, indicating that Cu_2_S/Pd/CuO has good electrical conductivity.

Immunosensors need to have good conductivity and catalysis, which are important for the collective effect of the various materials (Zheng et al., 2021). The sensitivity of the unlabeled immunosensor mainly depends on the reducibility of the constructed material to H_2_O_2_ (Yan et al., 2018). Chronoamperometry (*i*–*t*) can be used to compare the catalytic activities of different modified materials (Lee et al., 2018). As shown in Figure 4B, naked GCE has no catalytic activity for H_2_O_2_ (curve a). When Cu_2_S was loaded onto the electrode, the catalytic signal increased slightly (curve b); the signal of Cu_2_S/Pd (curve d) was greater than that of Cu_2_S (curve b). This is due to the better catalytic performance of Pd NPs and the large specific surface area of snowflake-like materials, which can support more Pd NPs. Cu_2_S generated Cu_2_S/CuO (curve c) in the presence of hydrogen peroxide, and its catalytic activity was enhanced. It was further found in this experiment that the catalytic activity of Cu_2_S/Pd/CuO (curve e) was higher than that of Cu_2_S/Pd (curve d), and the current response was more stable. Therefore, the Cu_2_S/Pd/CuO nanocomposite material was used as a signal-amplifying platform to construct a highly sensitive and unlabeled electrochemical immunosensor. After the modification of Cu_2_S/Pd/CuO on the GCE, the peak current after multiple scanning, calculated by measuring the CV response, was basically unchanged, indicating that the prepared nanocomposite has good stability (Figure 4C). The successful construction of the sensor was verified by EIS detection of layers of modification (Figure 4D and Supplementary Table S2). Compared with the bare GCE (curve a), the resistance of the Cu_2_S/Pd/CuO (curve b) electrode was higher. When anti-CEA (curve c), BSA (curve d), and CEA (curve e) were successively modified to the working electrode surface, the semicircle diameter of the electrical impedance spectra increased continuously, which was attributed to the partial inhibition of electron transfer by proteins (Supplementary Table S2), indicating that the electrochemical immunosensor was successfully modified.

### Optimization of the Experimental Conditions

In order to obtain the best measurement results for the tumor markers, experimental conditions such as the substrate concentration of Cu_2_S/Pd/CuO and the pH of PBS need to be optimized. The immunosensor was constructed based on a CEA concentration of 1 ng/ml in this study.

**FIGURE 5:**
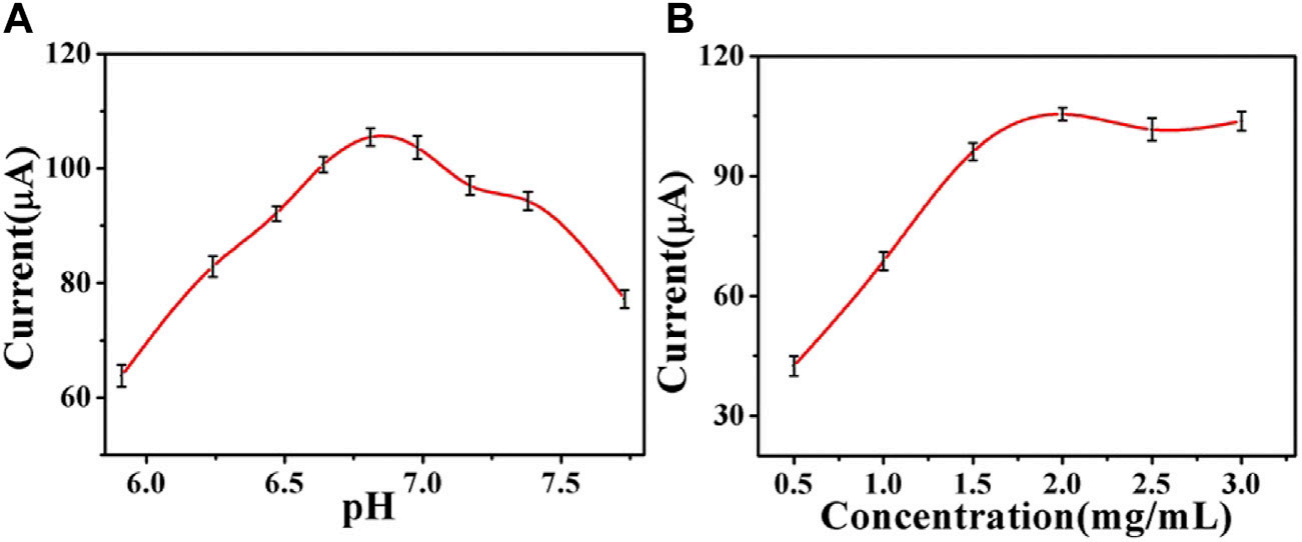
Optimization of the experimental conditions of the pH value **(A)** and Cu_2_S/Pd/CuO concentration **(B)**. Error bar = SD.

The pH value of PBS is important for the catalytic properties of the immunosensor because a strongly acidic or alkaline environment may inactivate antigens and antibodies, thus affecting the specificity of protein binding. When the pH of PBS changed from 5.91 to 6.81, the current signal began to increase to a peak. However, when the pH of PBS exceeded 6.81, the current response was reduced. Therefore, the maximum current signal was obtained at a pH of 6.81, which maintained good biological activity. Therefore, PBS with pH of 6.81 was selected as the best electrolyte for electrochemical measurements (Figure 5A). The concentration of Cu_2_S/Pd/CuO is one of the most important parameters affecting the performance of the electrochemical immunosensor. The concentration of Cu_2_S/Pd/CuO will have an impact on the electron transfer and the loading capacity of the anti-CEA. In order to obtain the best performance of the immunosensor, working electrodes with different concentrations of Cu_2_S/Pd/CuO were used, and 10 μl (5.0 mM) H_2_O_2_ was injected into 10 ml PBS at pH 6.81. As shown in Figure 5B, the current response increased significantly with the increase of Cu_2_S/Pd/CuO concentration from 0.5 to 2.0 mg/ml, and then decreased gradually with the increase of Cu_2_S/Pd/CuO concentration from 2.0 to 3.0 mg/ml. Therefore, the optimal concentration for the immunosensor construction in this study was 2.0 mg/ml.

### Performance Analysis of the Immunosensor

**FIGURE 6:**
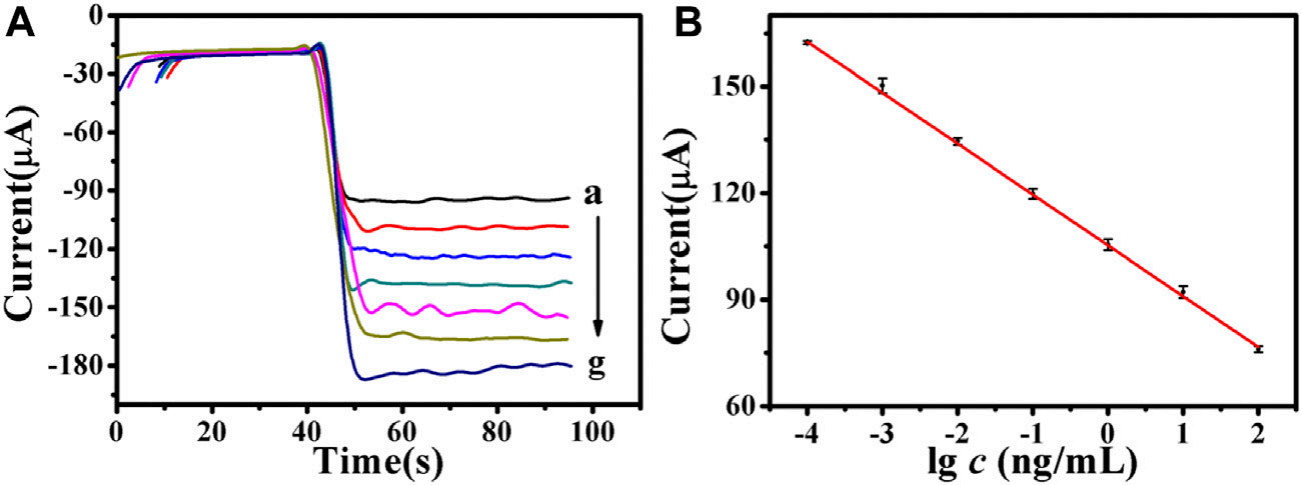
**(A)** Amperometric response of the electrochemical immunosensor to different concentrations of carcinoembryonic antigen (CEA), from *a* to *g*: 100 fg/ml, 1 pg/ml, 10 pg/ml, 100 pg/ml, 1 ng/ml, 10 ng/ml, 100 ng/ml, respectively. **(B)** Calibration curves of the immunosensor to different concentrations of CEA. Error bar = SD.

In this experiment, an electrochemical immunosensor with good conductivity and catalytic activity was prepared with the Cu_2_S/Pd/CuO composite material. A series of CEA concentrations were measured by chronoamperometry. With the increase of CEA concentration, the current signal of the immune sensor decreased, indicating that the antigen impeded the electron transport (Figure 6A). In the range from 100 fg/ml to 100 ng/ml, the linear regression equation of the CEA concentration to the value and current response was *Y* = −14.34lg*C* + 105.24, and the correlation coefficient was 0.9997 (Figure 6B). The limit of detection (LOD) was 33.11 fg/ml (S/N = 3).

Compared with other reported CEA results of the immunosensor (Supplementary Table S3), the immunosensor in this study showed a comparable linear range (from 100 fg/ml to 100 ng/ml) and an improved LOD (33.11 fg/ml). The reasons for the low detection limit of the prepared immunosensor were as follows: firstly, Cu_2_S/Pd/CuO has good biocompatibility, which ensures antigen activity and can effectively fix antibodies. Secondly, Cu_2_S/Pd/CuO can provide a wider detection range as a multi-signal amplification platform. Finally, Cu_2_S/Pd/CuO has good electron transfer performance, which benefits improving the sensitivity of the electrochemical immunosensor.

**FIGURE 7:**
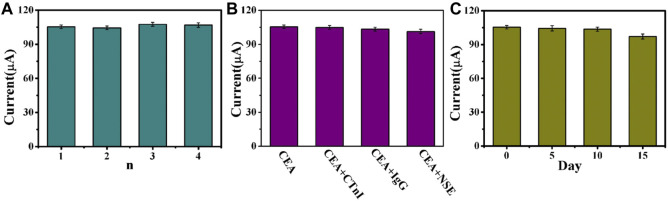
**(A)** Current change response of the biosensor to five different electrodes treated in the same process (carcinoembryonic antigen, CEA = 1 ng/ml). **(B)** Current signals of the compound of interfering substances (20 ng/ml) and CEA (1 ng/ml). **(C)** Stability study of the CEA immunosensor (CEA = 1 ng/ml). Error bar = SD.

After the electrochemical immunosensor was prepared, its performance needs to be verified, such as its repeatability, selectivity, and stability. These are important parameters for the evaluation of clinical application methods. Firstly, five immunosensors were prepared with the same concentration of CEA by chronoamperometry to detect the current signal; the repeatability of the electrochemical immunosensor was then studied. The relative standard deviation (RSD) of the electrode was calculated as 1.15%, indicating that the immune sensor has good repeatability (Figure 7A). Secondly, the selectivity of the immunosensor was studied using troponin I (CTnI), immunoglobulin G (IgG), and neuron-specific enolase (NSE) as the interfering substances. As shown in Figure 7B, the RSD was less than 1.79%, indicating that the selectivity of the immunosensor is relatively reliable. Finally, the stability of the immunosensor was assessed by measuring the current response of the five working electrodes and storing them in a 4C refrigerator when not tested once a day for five consecutive days. After 15 days, the current signal obtained decreased from 100% to 92.1% (Figure 7C), showing good stability. The experimental results showed that the immunosensor has good reproducibility, selectivity, and stability and can be used for highly sensitive and quantitative detection of CEA.

### Detection of CEA in Human Serum Samples

In order to evaluate the application potential and reliability of the immunosensor designed in this study, a standard recovery experiment and human serum sample test were conducted. The results showed that the recoveries were 95.5%–108%, as shown in Table 1. Compared with that of commercial electrochemiluminescence immunoassay (ECLIA), the relative error was 3.70%–5.89% (Supplementary Table S4), which proves the feasibility of the designed immunosensor. These results indicate that the constructed immunosensor has potential application value in clinical serum CEA detection.

**TABLE 1:** Detection of carcinoembryonic antigen (CEA) in human serum samples

CEA content in serum (ng/ml)	Addition content (ng/ml)	Detection value (ng/ml)	Recovery (%)
	1.00	3.86	108
2.58	5.00	7.33	96.7
	10.0	12.0	95.5

## Conclusion

We used Cu_2_S/Pd/CuO as the signal amplification platform to successfully prepare an unlabeled immunosensor that can detect CEA quantitatively and sensitively. The Cu_2_S/Pd/CuO sensing platform can effectively capture the substance to be measured, amplify the current signal, and improve the catalytic performance. The unlabeled immunosensor has good specificity, repeatability, and stability. At the same time, the detection limit of the electrochemical method is low and the linear range is wide, which meet the requirements of human serum sample detection. This strategy has important application value in the clinical diagnosis related to other molecular markers.

## Data Availability

The original contributions presented in the study are included in the article/Supplementary Material. Further inquiries can be directed to the corresponding author.
