# Supplementary material for: A Label-Free Electrochemical Immunosensor for CEA Detection on a Novel Signal Amplification Platform of Cu2S/Pd/CuO Nanocomposites
Source: Front Bioeng Biotechnol. 2021 Dec 10;9:767717. doi: 10.3389/fbioe.2021.767717 (PMC8702859; doi:10.3389/fbioe.2021.767717)
Supplement: Supplementary file 1 [file DataSheet1.docx]

**Electronic Supplementary Material**

A label-free electrochemical immunosensor for CEA detection on a novel signal amplification platform of Cu_2_S/Pd/CuO nanocomposites

**Linlin Cao^1, 3^, Wen Zhang ^3^, Sumei Lu ^1, 2^, Chengjie Guo^3^, Peijun Wang^1^, Dantong Zhang^2^, Wanshan Ma^1,2*^**

1. ***Department of Laboratory Medicine, Shandong Provincial Qianfoshan Hospital, Shandong University, Jinan, Shandong 250014, P. R. China***
2. ***Department of Laboratory Medicine, The First Affiliated Hospital of Shandong First Medical University, Jinan, Shandong 250014, P. R. China***
3. ***Zibo Central Hospital, Shandong University, Zibo, 255036, PR China***

* To whom correspondence should be addressed.

E-mail: [mwsqianyi@163.com](mailto:mwsqianyi@163.com)

**The calculation of LOD**

The definition of the limit of detection (LOD) was calculated according to the definition of LOD of the International Union of Pure and Applied Chemistry (IUPAC)[1]. The LOD was expressed by concentration, which refered to the lowest concentration (*c_L_*) obtained from the minimum analysis signal (*x_L_*) that could be reasonably detected by a specific analysis step. When we calculated the LOD of the sensor, we measured the blank sample 7 times, obtained the average value (*x_b1_*) and standard deviation (*s_b1_*) of the electrochemical signal corresponding to the blank sample, and calculated that the minimum electrochemical signal detected should be *x_L_ = x_b1_ + k s_b1_*. In the formula, *x_b1_* was the average value of the blank sample; *s_b1_* was the standard deviation of the blank sample; *k* was a numerical factor selected according to the desired confidence level. IUPAC recommended *k* = 3 as the detection limit calculation standard, and the corresponding confidence was about 90%.

In this work, after taking ten blank measurements, the *x_b1_* = 166.23 and *s_b1_* = 1.0828 was obtained. Therefore, *x_L_ = x_b1_ + k s_b1_* = 166.23 + 3 × 1.0828 = 169.48. The calibration plot of this proposed immunosensor is *I* (µA) = -14.34 lg *c* (ng/mL) + 105.24. Therefore, the *c_L_* = 10 ^(169.48 – 105.24) / (-14.34)^ = 3.311 × 10 ^-5^ ng/mL = 33.11 fg/mL[2]. In conclusion, the LOD of this proposed immunosensor is 33.11 fg/mL.

**Table S1** The R_et_ of nanocomposite EIS.

| Electrode | Resistance (Ω) |
| --- | --- |
| Cu_2_S/GCE (a) | 2550 |
| Cu_2_S/Pd/GCE (b) | 1158 |
| Cu_2_S/Pd/CuO/GCE (c) | 1237 |

**Table S2** The R_et_ of EIS after layer construction of immunosensor.

| Electrode | Resistance (Ω) |
| --- | --- |
| GCE (a) | 400.2 |
| Cu_2_S/Pd/CuO/GCE (b) | 1237 |
| Ab_1_/Cu_2_S/Pd/CuO/GCE (c) | 2193 |
| BSA/Ab_1_/Cu_2_S/Pd/CuO/GCE (d) | 3081 |
| CEA/BSA/Ab_1_/Cu_2_S/Pd/CuO/GCE (e) | 4288 |

**Table S3** Methodology comparison of CEA detection by different immunosensors.

| Signal amplification platform | Detection method | Detection range | Detection limit | Reference |
| --- | --- | --- | --- | --- |
| TiO2/DS-ZnCdS | Photoelectrochemical immunosensor | 0.001-50 ng /mL | 0.1 pg/mL | [3] |
| Ni/C@SiO2–NH2 | Sandwich-type electrochemical immunosensor | 0.006-12.00ng/mL | 1.56 pg/mL | [4] |
| Au@Ce2Sn2O7 | Label-free electrochemiluminescence immunosensor | 0.001-70 ng/mL | 0.53 fg/mL | [5] |
| Polyaniline@Au | Photothermal immunoassay | 0.20-25ng/mL | 170 pg/mL | [6] |
| reduced graphene oxide | label-free electrochemical immunosensor | 0.1-5 ng/mL | 50 pg/mL | [7] |
| FTO/g-C3N4/CdSe electrode | Label-free photoelectrochemical immunosensor | 10-100000 ng/mL | 210 pg/mL | [8] |
| Sulfur-doped graphene sheet (S-GS) | The multiplex immunoassay | 0.0001-0.3 ng/mL | 30 fg/ml | [9] |
| GO | UC-FRET aptasensor | 0.03-6 ng/mL | 7.9 pg/mL | [10] |
| Ti_3_C_2_-MXene/AuNPs | Surface plasmon resonance sensors | 0.0002-20000 pM | 0.07 fM | [11] |
| Cu_2_S/Pd/CuO | Label-free electrochemical immunosensor | 0.0001-100 ng/mL | 33.11 fg/mL | This work |

**Table S4** Human serum sample analysis by immunosensor and the ECLIA method.

| Sample number | ECLIA (ng/mL) | Immunosensor (n=3) (ng/mL) | Relative error (%) |
| --- | --- | --- | --- |
| 1 | 8.66 | 8.15±0.79 | 5.89 |
| 2 | 1.08 | 1.12±0.08 | 3.70 |
| 3 | 4.99 | 4.73±0.58 | 5.21 |

**Reference**

[1] V. Fassel, Nomenclature, symbols, units and their usage in spectrochemical analysis—II. data interpretation Analytical chemistry division, Spectrochimica Acta Part B: Atomic Spectroscopy 33(6) (1978) 241-245.

[2] K. Ren, J. Wu, F. Yan, Y. Zhang, H. Ju, Immunoreaction-triggered DNA assembly for one-step sensitive ratiometric electrochemical biosensing of protein biomarker, Biosensors and Bioelectronics 66 (2015) 345-9.

[3] Y. Zang, R. Cao, C. Zhang, Q. Xu, Z. Yang, H. Xue, Y. Shen, TiO2-sensitized double-shell ZnCdS hollow nanospheres for photoelectrochemical immunoassay of carcinoembryonic antigen coupled with hybridization chain reaction-dependent Cu(2+) quenching, Biosens Bioelectron 185 (2021) 113251.

[4] D. Song, J. Zheng, N.V. Myung, J. Xu, M. Zhang, Sandwich-type electrochemical immunosensor for CEA detection using magnetic hollow Ni/C@SiO2 nanomatrix and boronic acid functionalized CPS@PANI@Au probe, Talanta 225 (2021) 122006.

[5] M.S. Khan, H. Ameer, Y. Chi, Label-Free and Ultrasensitive Electrochemiluminescent Immunosensor Based on Novel Luminophores of Ce2Sn2O7 Nanocubes, Anal. Chem. 93(7) (2021) 3618-3625.

[6] B. Zhang, X. Hu, Y. Jia, J. Li, Z.J.M.a. Zhao, Polyaniline@Au organic-inorganic nanohybrids with thermometer readout for photothermal immunoassay of tumor marker, 188(3) (2021) 63.

[7] M. Jozghorbani, M. Fathi, S.H. Kazemi, N. Alinejadian, Determination of carcinoembryonic antigen as a tumor marker using a novel graphene-based label-free electrochemical immunosensor, Anal. Biochem. 613 (2021) 114017.

[8] X.P. Liu, J.S. Chen, C.J. Mao, B.K. Jin, A label-free photoelectrochemical immunosensor for carcinoembryonic antigen detection based on a g-C3N4/CdSe nanocomposite, Analyst 146(1) (2021) 146-155.

[9] X. Ren, H. Ma, T. Zhang, Y. Zhang, T. Yan, B. Du, Q. Wei, Sulfur-Doped Graphene-Based Immunological Biosensing Platform for Multianalysis of Cancer Biomarkers, ACS Appl Mater Interfaces 9(43) (2017) 37637-37644.

[10] Y. Wang, Z. Wei, X. Luo, Q. Wan, R. Qiu, S. Wang, An ultrasensitive homogeneous aptasensor for carcinoembryonic antigen based on upconversion fluorescence resonance energy transfer, Talanta 195 (2019) 33-39.

[11] Q. Wu, N. Li, Y. Wang, Y. Liu, Y. Xu, S. Wei, J. Wu, G. Jia, X. Fang, F. Chen, X. Cui, A 2D transition metal carbide MXene-based SPR biosensor for ultrasensitive carcinoembryonic antigen detection, Biosens Bioelectron 144 (2019) 111697.
